# Inhalation of carbon monoxide is ineffective as a long-term therapy to reduce obesity in mice fed a high fat diet

**DOI:** 10.1186/2052-9538-1-6

**Published:** 2014-03-04

**Authors:** Peter A Hosick, Elhaitham K Ahmed, Monette U Gousset, Joey P Granger, David E Stec

**Affiliations:** Department of Physiology & Biophysics, Center for Excellence in Cardiovascular-Renal Research, University of Mississippi Medical Center, 2500 North State St, Jackson, MS 39216 USA

**Keywords:** Heme oxygenase, Obesity, Type II diabetes, Metabolism, Insulin resistance

## Abstract

**Background:**

Previous studies have demonstrated that induction of heme oxygenase-1 results in weight loss in several rodent models of obesity. However, the specific role of the heme oxygenase-1 metabolite, carbon monoxide (CO), in this response has yet to be established. We recently reported that chronic treatment with CO releasing molecules results in prevention of weight gain in mice fed a high fat diet. In the present study, we sought to determine the effect of chronic CO inhalation on the development and reversal of high fat diet induced obesity.

**Results:**

CO inhalation at both levels initially resulted in a prevention and reversal of body weight and fat mass over the first 10 weeks of treatment, however, this effect was not sustained. CO inhalation in the prevention groups also had an early effect to lower fasting blood glucose but this effect also was not sustained.

**Conclusions:**

Our results demonstrate that CO inhalation has a transient effect to prevent and reduce body weight which is not sustained chronically in mice fed a high fat diet. These results suggest that chronic CO inhalation therapy is not an effective treatment to induce long term weight loss.

## Background

Despite a concerted effort to educate the public about the problem of obesity, rates continue to increase worldwide. As the problem of obesity expands so does the associated condition of metabolic syndrome which is characterized by insulin resistance, dyslipidemia, reduced HDL cholesterol, and hypertension. These disorders increase the risk for development of several metabolic and cardiovascular diseases such as type II diabetes, coronary artery disease, stroke, and end-stage renal disease [1, 2]. Exploring potential treatment options for prevention and reversal of obesity and its associated diseases is paramount to finding a long term solution to this worldwide problem. Carbon Monoxide (CO) is endogenously produced in the body via the catabolism of heme by heme oxygenase (HO) enzymes and by oxidation of lipids where it serves as a gaseous transmitter [3–5]. HO enzymes exist in two major isoforms: heme oxygenase-1 (HO-1) and heme oxygenase-2 (HO-2). HO-1 is a 32 kilodalton protein which is inducible by a wide variety of stimuli including oxidants, metals, and hypoxia [6]. HO-2 is a 36 kilodalton protein which is the constitutively expressed isoform important in vascular function [7, 8]. In addition to endogenously produced CO, combustion of carbon compounds produces colorless odorless CO gas. CO can be poisonous when levels greater than 1500 parts per million (ppm) in the air are inhaled as CO has a greater affinity for hemoglobin then does oxygen ultimately resulting in severe tissue hypoxia.

Recent studies have demonstrated the beneficial actions of chronic low dose CO inhalation for the prevention of ischemic kidney injury and the lowering of blood pressure [9–11]. Beneficial actions of CO inhalation were found in the range between 20-250 ppm which are several fold lower than the levels at which CO becomes poisonous. Numerous studies have demonstrated that chemical induction or genetic overexpression of HO-1 can attenuate the development of obesity establishing a possible role of endogenously produced CO to attenuate the development of obesity [12–15]. While the HO-1 enzyme system has been established to reduce weight gain in obesity the role of HO metabolite CO has received less attention for its role in obesity prevention. Recently our lab demonstrated that chronic treatment with CO releasing molecules (CORMs), which release CO independent of HO-1 induction, prevent the development of obesity in mice fed a high fat diet [16]. Based upon our findings that chronic treatment with CORMs attenuates weight gain in mice fed a high fat diet, we sought to determine if CO inhalation would have a similar effect as CORM injection in the prevention of obesity in response to a high fat diet. In the present study, 4 groups of mice on a high fat diet were placed in a CO inhalation chamber daily for 30-weeks to determine the specific effect of chronic CO inhalation on either the development or reversal of dietary induced obesity.

## Methods

### Animals

The experimental procedures and protocols of this study conform to the National Institutes of Health Guide for the Care and Use of Laboratory Animals and were approved by the Institutional Animal Care and Use Committee of the University of Mississippi Medical Center.

The ability of CO inhalation to either prevent or reverse obesity was studied using separate groups exposed to 2 different concentrations of CO. CO inhalation was performed in a specially designed chamber which allowed mice to be exposed to different levels of CO without leaving their home cages. Two different levels of CO inhalation exposures were used in the present study. Mice were exposed to CO at 28 parts per million (ppm) for 2 hours daily (CO-28) or 200 ppm CO for 1 hour daily (CO-200). All mice were housed under standard conditions with full access to a 60% high fat diet (diet # D12492, Research Diets, Inc., New Brunswick, NJ) and water. Control mice were fed the high fat diet but did not did not receive any CO exposure. For the prevention study, a separate cohort of mice was maintained on a 17% normal fat diet (Teklad 22/5 rodent diet, #8640, Harland Laboratories, Inc., Indianapolis, IN).

### CO inhalation

Mice were exposed to CO in a 25 × 27 × 38 cm clear plastic chamber. A mixture of CO and air was flushed through the chamber at a rate of 0.05–30 l/min. Pure CO gas was mixed with appropriate amounts of air to dilute the CO to the desired concentration in parts per million (ppm), either ~28 or 200 ppm CO. A sampling line was fed from the chamber and passed through a Grey Wolf Indoor Air IQ-410 CO gas analyzer (Shelton, CT) which was connected to a lap-top computer and gave real time CO gas measurements using the WolfSense software package (Version 2011.28, Shelton, CT). The CO concentration was verified using a handheld CO meter placed inside the inhalation chamber (C.E., China). This setup allowed for mice to be exposed to the different levels of CO with free access to food and water in their home cages throughout the daily treatment periods. Mice were exposed to CO in the mornings for 2 hours in mice receiving 28 ppm CO and for 1 hour in mice receiving 200 ppm.

### Carboxyhemoglobin (COHb) measurement

Using a separate group of mice from those used in the chronic CO inhalation studies; COHb was measured before, immediately after and at 30, 60 and 90 minutes following each CO inhalation exposure protocol. Blood was collected via the orbital sinus under light isoflurane anesthesia. Immediately after collection, the blood sample was analyzed for COHb levels using Radiometer ABL80 Flex CO-OX analyzer (Westlake, OH) which requires 105 μl of whole blood. COHb levels are expressed as a percentage of total hemoglobin.

### Body composition (EchoMRI)

Body composition of all mice was assessed at 6 week intervals throughout the study using magnetic resonance imaging (EchoMRI-900TM, Echo Medical System, Houston, TX). EchoMRI measurements were performed in conscious mice placed in a thin-walled plastic cylinder with a cylindrical plastic insert added to limit movement of the mice. Mice were briefly submitted to a low intensity electromagnetic field allowing both fat mass and lean mass to be measured.

### Fasting glucose

Following an overnight fast (~16 hours) a blood sample was obtained under isoflorane anesthesia via orbital sinus. Blood glucose was measured using an Accu-Chek Advantage glucometer (Roche, Mannheim, Germany).

Oxygen consumption, carbon dioxide production and motor activity. Twenty-eight weeks after the initiation the experimental protocol 4 mice from each group were placed individually in an acrylic cage (16 cm × 24 cm × 17 cm) equipped with a metabolic monitoring system (AccuScan system, Harvard Apparatus, Holliston, Massachusetts) for measurements of oxygen consumption (VO_2_), carbon dioxide production (VCO_2_) and respiratory quotient (RQ) as previously described [15, 16]. VO_2_, VCO_2_ and RQ were determined daily (for 2 min every 10-min interval) and expressed as the 24 hour average. RQ was calculated by the formula: VCO_2_/VO_2_. Motor activity was determined using infrared light beams mounted in the cages in *x*, *y*, and *z* axes. Heat production was derived from the following formula (4.33 + (0.67*RQ)*VO_2_*weight (g)*60). After the mice were acclimatized to the new environment for 1 day, VO_2,_ VCO_2_, RQ and animal activity were recorded for 2 consecutive days.

### Glucose tolerance test

For glucose tolerance tests (GTT), mice were subjected to an overnight fast (∼16 h) and intraperitoneal (IP) injection of D-glucose (1 g/kg of body weight). Blood glucose was monitored at 0, 15, 30, 60, and 90 min after glucose injection using a portable glucose analyzer (Accu-Chek Advantage glucometer, Roche, Mannheim, Germany).

### Food consumption

Food consumption was measured at two time points, early (10 weeks) and late (28 weeks) during the 30 weeks of inhalation treatment. The total amount of food was weighed daily in the morning for 5 consecutive days. The daily consumption over the 5 day period was averaged for each mouse to obtain 24-hour food consumption.

### Western blot analysis

Western blots were performed on lysates prepared from tissues collected at the end of the experiments. Samples of 30 μg of protein were boiled in Laemmli sample buffer (Bio-Rad, Hercules, CA) for 5 min and electrophoresed on 10 or 12.5% SDS-polyacrylamide gels and blotted onto nitrocellulose membrane. Membranes were blocked with Odyssey blocking buffer (LI-COR, Lincoln, NE) for 2 hours at room temperature and then incubated with primary antibodies overnight at 4°C. Membranes were incubated with either Alex 680 (Molecular Probes) or IR Dye 800 (Rockland, Gilbertsville, PA) secondary antibodies for 1 hour at room temperature. Membranes were visualized using an Odyssey infrared imager (Li-COR, Lincoln, NE) which allows for the simultaneous detection of two proteins. Densitometry analysis was performed using Odyssey software (LI-COR, Lincoln, NE). Antibodies for Western blots were as follows: aldehyde dehydrogenase-1 (ALDH1A1; Abcam, Cambridge, MA), HO-1 (Enzo Life Sciences, Farmingdale, NY), nuclear receptor factor-1 (NRF-1; Rockland, Gilbertsville, PA), peroxisomal proliferating activating receptor-γ coactivator (PGC1-α; Cell Signaling Tech, Boston, MA), uncoupling protein-1 (UCP-1; Sigma, St. Louis, MO) and β-actin (Abcam, Cambridge, MA). All antibodies were used at a ratio of 1:1000 with blocking buffer, the lone exception being β-actin which was used at a ratio of 1:5000. All blots from tissue samples were run in duplicate with 3 samples from each group per gel with prevention reversal samples run on separate gels. These proteins were selected based upon our previous study which demonstrated that these proteins are regulated by CO [16].

### Statistics

All data are presented as mean ± s.e.m. Differences between treatment groups were determined using one-way analysis of variance with a *post hoc* test (Dunnett’s). A *P* < 0.05 was considered to be significant. All analyses were performed with SigmaStat (Systat software Inc., Richmond, CA, USA).

## Results

### Co inhalation and blood carboxyhemoglobin levels

**Figure 1:**
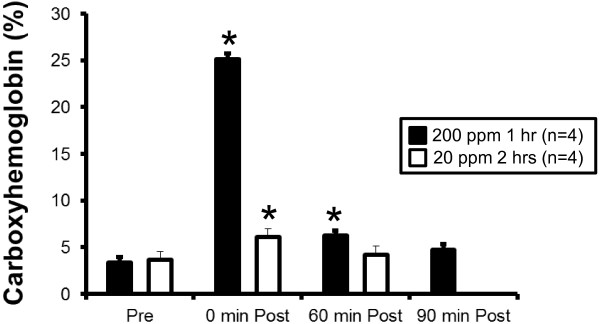
**Carboxyhemoglobin percentage before and following inhalation of CO at 200 ppm for 1 hr (n = 4) or 20 ppm for 2 hrs (n = 4).** * = significant from pre value, p < 0.05.

The time course of CO inhalation on blood carboxyhemoglobin (COHb) levels in response to inhalation of 28 or 200 ppm CO was examined in separate groups of mice. CO inhalation of 28 ppm for 2 hours resulted in significant doubling of blood COHb levels which returned to baseline levels 60 min after being removed from CO inhalation chamber (Figure 1). CO inhalation of 200 ppm CO for 1 hour resulted in an increase in blood COHb levels by 8 fold which were still significantly elevated from baseline 60 min after inhalation but returned to baseline levels by 90 min post inhalation (Figure 1). These dissociation profiles are in agreement with a previous study which examined the effects of high levels (500 ppm) CO inhalation in mice [17].

### Chronic CO inhalation transiently reduces body mass and fat

**Figure 2:**
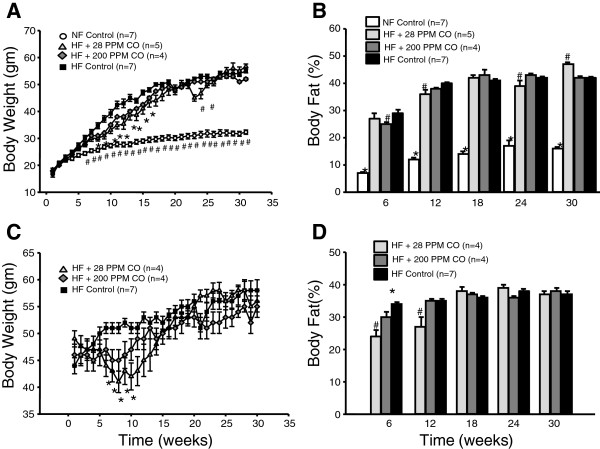
**Effect of chronic CO inhalation on: (A) obesity prevention body weight measured weekly, (B) obesity prevention body fat percentage as measured by echo-MRI, (C) obesity reversal body weight measured weekly, (D) obesity reversal body fat percentage as measured by echo-MRI.** * = significant from HF Control, *p* < 0.05. # = significant from all other groups, p < 0.05.

In the obesity prevention groups, high fat diet resulted in elevations in body weight by week 6 of treatment compared to mice fed a normal 17% fat mouse chow (Figure 2A). Both CO inhalation groups exhibited a reduction of body weight after 8 weeks of treatment which remained until week 16 of treatment at which point body weight was no different from control high fat diet mice (Figure 2A). CO-28 mice had a significant reduction in body weight as compared to control high fat mice at weeks 23 and 24; however, body weight increased to similar levels at week 25 and remained similar for the duration of the study (Figure 2A). Body fat percentage was significantly reduced in the normal diet control prevention mice compared to all high fat diet mice at all time points measured (Figure 2B). While body weight was significantly reduced at 8 weeks into treatment in the CO-28 mice, body fat percentage as measured by EchoMRI was not significantly reduced until 12 weeks into treatment (Figure 2B). At week 18 of the study, body fat percentage was not different in the CO-28 mice; however, it was significantly reduced by 24 weeks (Figure 2B). Surprisingly, body fat percentage was significantly increased in the CO-28 mice as compared to the other groups of high fat fed mice at week 30 (Figure 2B). Body fat percentage in the CO-200 treatment group was significantly reduced compared to the high fat control group at only week 6 of treatment and then remained at similar levels for the duration of the study (Figure 2B).

In the obesity reversal groups, both CO inhalation groups exhibited a significant reversal of weight gain in the first 5-10 weeks of treatment (Figure 2C). However, this effect was lost after week 10 and their body weights returned to similar levels as compared to the control high fat diet group (Figure 2C). Body fat percentage was significantly reduced in the CO-28 group at weeks 6 and 12 into treatment; whereas, the CO-200 group was significantly reduced at week 6 only (Figure 2D). There was no significant difference in body fat mass between the groups for the remainder of the obesity reversal study (Figure 2D).

### Chronic CO inhalation transiently reduces blood glucose levels

**Figure 3:**
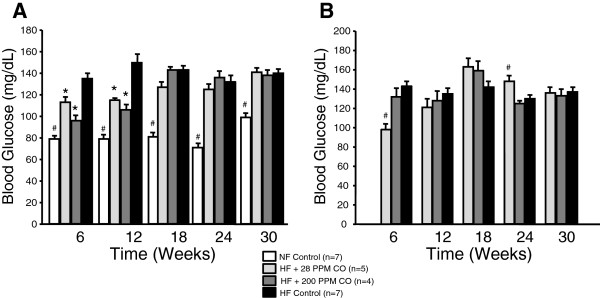
**Effect of chronic CO inhalation on fasting blood glucose in the: (A) Prevention groups, (B) Reversal groups.** * = significant from HF Control, p < 0.05. # = significant from all other groups, *p* < 0.05.

In the obesity prevention groups, both the CO-28 and CO-200 groups had significantly reduced blood glucose compared to the high fat diet control mice at 6 and 12 weeks into treatment but this effect was lost was lost at 18, 24, and 30 weeks (Figure 3A). Normal diet control mice had significantly reduced blood glucose levels from all other groups at each time point measured (Figure 3A). The CO-28 reversal obesity mice had significantly reduced blood glucose level at 6 weeks, however; by week 24 these mice had blood glucose levels significantly elevated compared to control mice (Figure 3B). Fasted blood glucose of the CO-200 mice was not different from the HFD control mice at any time point measured (Figure 3B).

### Chronic treatment with CO has no long term effect on oxygen consumption, carbon dioxide production, motor activity, heat production, or food intake

In obesity prevention groups, no significant differences existed in VO_2_, VCO_2_, or Respiratory Exchange Ratio (RER) between any of the groups (Table 1). Heat production was greater in all HFD groups compared to normal diet controls (p < 0.05) whereas motor activity was reduced in all HFD groups compared to normal diets controls (p < 0.05) (Table 1). Finally, the normal weight group had significantly reduced food intake compared to the HF control group at week 10 of the treatment (p < 0.05) (Table 1). In the obesity reversal groups there was no significant difference between the groups with respect toward VO_2_, VCO_2_, RER, heat production, motor activity, or food intake at either time point measured (Table 1).

**Table 1:** **Average oxygen consumption (VO**_**2**_ **), carbon dioxide production (VCO**_**2**_ **), respiratory exchange ratio (RER), heat production, motor activity, food consumption at week 10 of treatment and food consumption at week 28 of treatment**

	Prevention				Reversal		
	HFD	HFD	HFD	Normal	HFD	HFD	HFD
	CO-28	CO-200	Control	Control	CO-28	CO-200	Control
	(n = 4)	(n = 4)	(n = 4)	(n = 4)	(n = 4)	(n = 4)	(n = 4)
VO_2_ (ml/kg/min)	54.2 ± 8.2	50.3 ± 8.1	55.6 ± 2.8	61.5 ± 5.7	56.5 ± 12.4	55.8 ± 8.8	52.7 ± 8.7
VCO_2_ (ml/kg/min)	40.3 ± 6.5	37.0 ± 7.3	40.7 ± 2.4	46.0 ± 4.4	41.2 ± 9.6	40.7 ± 6.5	39.1 ± 7.5
RER	0.73 ± 0.02	0.73 ± 0.04	0.73 ± 0.02	0.74 ± 0.02	0.73 ± 0.03	0.73 ± 0.01	0.74 ± 0.02
Heat Prod. (Cal/hr)	826 ± 135	797 ± 105	857 ± 43	605 ± 68*	828 ± 168	912 ± 158	883 ± 84
Motor Act. (m/day)	3.88 ± 0.90	3.39 ± 0.56	4.44 ± 1.84	8.87 ± 1.51*	4.52 ± 2.15	6.76 ± 3.24	3.77 ± 2.94
Food 10 weeks (g/day)	2.8 ± 0.1	2.5 ± 0.2	2.2 ± 0.1#	3.2 ± 0.8	3.7 ± 0.4	3.0 ± 0.1	3.2 ± 0.4
Food 28 weeks (g/day)	3.0 ± 0.2	3.0 ± 0.4	3.3 ± 0.8	3.4 ± 0.3	3.2 ± 0.2	3.4 ± 0.3	3.2 ± 0.3

Data are mean ± SD. *Significant from all HFD groups, p < 0.05. #Significant for normal diet control, p < 0.05.

### Chronic treatment with CO ameliorates the high fat diet induced increase in HO-1 in epididymal adipose tissue

**Figure 4:**
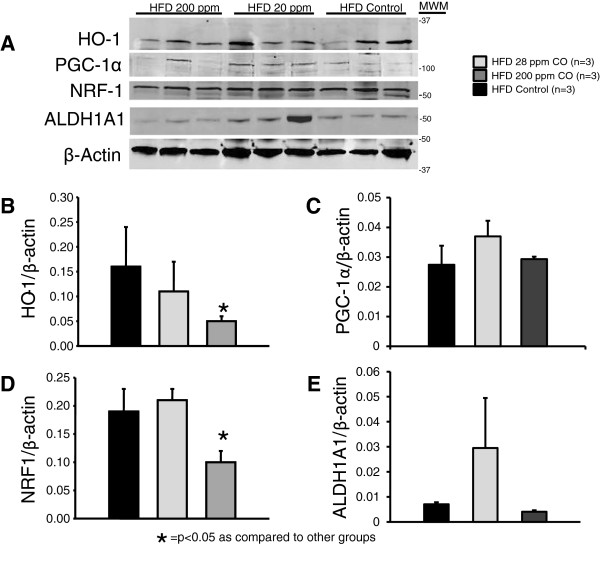
**Representative Western blots from epidydmal fat tissues of chronic CO inhalation for the reversal of obesity from high fat diet (HFD) control, HFD 28 ppm CO, HFD 200 ppm CO mice. A)** Representative blots. **B)** Levels of HO-1. **C)** Levels of PGC1-α. **D)** Levels of NRF1. **E)** Levels of ALDH1A1. * = significant from all other groups, *p* < 0.05. MWM = molecular weight marker.

In obesity reversal groups, HO-1 in epididymal adipose tissue was reduced by CO treatment: however, the reduction was only significant in the CO-200 mice (p < 0.05) (Figure 4). In obesity prevention groups, all HFD groups had increased HO-1 protein in epididymal adipose tissue compared to the normal diet group (p < 0.05) (Figure 4). Both CO inhalation groups had significantly reduced HO-1 compared to the high fat diet group.

### Chronic treatment with CO has differential effects on markers of mitochondrial biogenesis in epididymal adipose tissue

**Figure 5:**
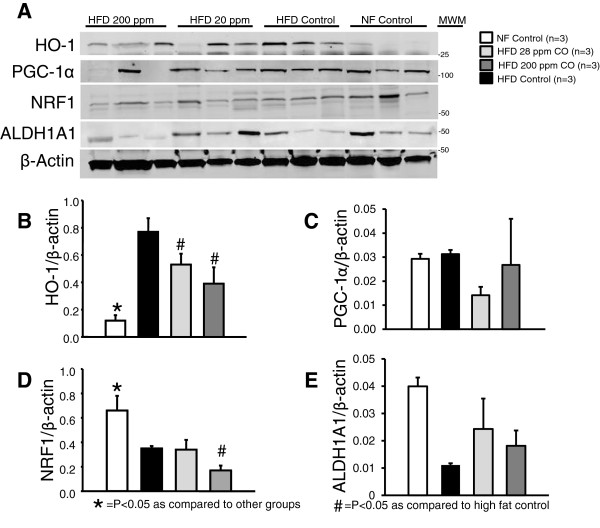
**Representative Western blots from epidydmal fat tissues of chronic CO inhalation for the prevention of obesity from normal fat control, high fat diet (HFD) control, HFD 28 ppm CO, HFD 200 ppm CO mice. A)** Representative blots. **B)** Levels of HO-1. **C)** Levels of PGC1-α. **D)** Levels of NRF1. **E)** Levels of ALDH1A1. * = significant from all other groups, *p* < 0.05. # = significant from HF Control mice, p < 0.05. MWM = molecular weight marker.

High fat diet reduced the marker of mitochondrial biogenesis NRF-1 (p < 0.05) in the prevention group. CO-28 treatment in the prevention groups had and additional effect to reduce the level of NRF-1 (p < 0.05) (Figure 5). The HFD Control group had a trend towards a reduction in the mitochondrial inhibitor ALDH1A1 (p = 0.065) whereas there was no trend towards reduced ALDH1A1 in either CO inhalation group (Figure 5). In the obesity reversal groups, NRF-1 was reduced by CO-28 treatment compared to both other groups (p < 0.05) (Figure 4).

## Discussion

Induction of HO-1 has been demonstrated to prevent the development of obesity in several rodent models [12–15]. However, the specific role of its metabolite, CO, in this response has not been established. We recently reported that chronic treatment with a CO releasing molecule, CORM-A1, prevented obesity in mice fed a high fat diet by increasing O_2_ consumption, heat production, and increased proteins in epididymal adipose tissue related to browning of adipose tissue [16]. In that study, CORM-A1 was administered by IP injection at a dose that does not increase blood COHb levels [16]. In the present study, we sought to determine if administration of CO via inhalation would have similar effects on both the development and maintenance of high fat diet induced obesity via alterations in O_2_ consumption, heat production, and remodeling of epididymal adipose tissue. Our results demonstrate that chronic inhalation of CO at either 200 ppm for 1 hour a day or 28 ppm for 2 hours a day resulted in a transient effect to prevent and reverse the progression of obesity in mice fed a high fat diet. This effect started at week 5 of treatment at lasted for 10 weeks in the prevention and 5 weeks in the reversal mice. During this time frame, mice in each treatment protocol exhibited reduced body weights compared to control mice fed a high fat diet despite eating similar amounts of food (Table 1). Thus, the reduced body weight in the treatment mice is not believed to be due to alterations in food consumption or interruption of the circadian rhythm. During this time frame, mice were protected against the development of type II diabetes and also exhibited decreased body fat percentage as compared to control mice fed the high fat diet alone. Both the effects on fasting blood glucose levels as well as body fat exhibited in mice receiving CO inhalation were lost after a 10 week period in the prevention mice and a 5 week period in the reversal mice. Interestingly, obesity prevention mice that received the CO-28 treatment went on to exhibit a greater body fat percentage following 30 of treatment as compared to the other groups on the high fat diet. While the mechanism responsible for the paradoxical nature of this chronic effect is not known, the level of CO exposure in the CO-28 mice is roughly equivalent to that found in cigarette smoke and may be a mechanism by which chronic smoking increases body weight. The paradoxical nature of CO-28 inhalation on body weight in response to a high fat diet needs to be examined in greater detail in future studies.

At present a full explanation for the transient nature of the effect of CO inhalation on body weight in mice fed a high fat diet is unknown. One potential explanation for the early weight loss in response to CO inhalation could be due to partial hypoxia especially in the mice receiving 200 ppm CO as this resulted in an 8 fold increase in blood COHb levels. This response could be a similar to the effect observed in sojourning to high altitudes and experiencing weight loss [18]. This altitude response is widely believed to be due to the development of anorexic conditions and can be avoided by maintaining adequate caloric intake [19, 20]. Several questions regarding the application of this hypothesis to the early effect observed in our CO inhalation groups exist. First, if hypoxic conditions were causing the prevention and reversal of weight loss we would have expected to see a more dramatic response in the CO-200 mice. As these mice were experiencing a greater level of hypoxia due to a more dramatic increase in blood COHb levels; however, that was not the case, in fact the opposite was true. Second, we measured food intake early in the treatment period during the time when the CO inhalation mice had significantly lower body weights compared to control mice. At this point there was no difference in food intake between any of the HFD groups (Table 1). Since hypoxia mediated weight loss is characterized by anorexic conditions, we do not believe that hypoxia in the CO inhalation mice explains the transient effect of weight loss observed in the present study.

A lack of a long term effect could be explained by an inability of CO to fully access adipose tissue and stimulate the browning of adipose tissue. This effect of CO on visceral and epididymal adipose tissue was observed in our previous study in which CORM-A1 was administered chronically via IP injection [16]. The mechanism of CO release into the tissue following inhalation is thought to be the same as for oxygen except the affinity of hemoglobin for CO is approximately 230 times that of oxygen. This extremely high affinity of CO for hemoglobin is what causes such severe hypoxia with CO poisoning. Once CO becomes bound to hemoglobin it is difficult to remove; thus, it may not be delivered to peripheral tissues, such as adipose tissue, as effectively as CO released from CORMs administered directly into the peritoneal cavity. It is possible that inhaled CO, which became tightly bound to hemoglobin, was circulated in the blood then became dissociated and exhaled. If this occurs the majority of the CO never would have reached the adipose tissue to function as an intracellular signaling molecule as would be the case when injected or produced endogenously via the HO enzyme system. Therefore, it is possible that the CO inhalation did not have a lasting chronic effect because inhalation of the gas is not an ideal delivery method to increase adipose CO levels chronically. The results from our Western blot experiments would support this hypothesis as no significant differences in markers of increased oxidative metabolism were present in the CO inhalation mice at 30 weeks of treatment. It is also possible that a different CO inhalation regimen might result sustained weight loss. Zheng et al. recently reported that CO inhalation at 250 ppm for 2 hours daily for 10 weeks resulted in significant weight loss, a decrease in adiposity, and a decrease in food intake measured at the end of the 10 week period [21]. This profile of weight loss is in agreement with the initial weight loss that we observed in our current study. However, whether CO inhalation of 250 ppm for 2 hours daily results in a sustained weight loss over a similar 30 week time frame was not determined in that study [21]. Since CO inhalation of 250 ppm for 2 hours was associated with a decrease in food intake when measured at 10 weeks, it is possible that observed weight loss in this model is a result of hypoxia induced anorexic conditions as previously mentioned. If this were the case the results of Zheng et al. [21] would be different from the observed weight loss in our current study, as we did not detect any effect of CO inhalation on food intake measured at either 10 or 28 weeks.

The mechanism responsible for the initial weight loss in the CO inhalation groups is not known at the present time. We did not perform any studies measuring O_2_ consumption, heat production, or activity at this early time point. One potential mechanism for the initial weight loss in the CO inhalation group is alteration in the circadian rhythm due to placement in the inhalation chambers. While this is a possibility, mice were placed in the inhalation chambers at the same time each morning about 1 hour after daylight and they were exposed in their home cages with free access to food and water. We did measure food intake early on and did not find any significant difference in the food intake of the any of the groups of mice on a high fat diet suggesting that alterations in food intake at this early time point does not account for the differences in body weight observed in the CO inhalation mice. The apparent trend for CO-28 inhalation to have a greater effect to reduce weight gain early on could possibly be due a greater effect on mitochondrial biogenesis and remodeling of adipocytes both of which could act to increase metabolism and promote weight loss in these mice [16]. It is also possible that this lower level of CO inhalation had a greater central effect to stimulate metabolism through an unidentified pathway; however, without any metabolic data specifically from this early time period the effect of this level of CO inhalation on metabolism is not known. At the end of the 30 week treatment period, we did not observe any changes in proteins such as PGC-1α, NRF-1, and ALDH1A1 in the mice inhaling CO suggesting that any effect CO inhalation may have had on mitochondrial biogenesis or remodeling of adipocytes was not sustained. Previous studies with the central administration of the HO-1 inducer, cobalt protoporphyrin (CoPP), have demonstrated an initial decrease in body weight that was associated with a decrease in food intake [22, 23]. However, while food intake eventually returns to normal levels, body weight is maintained at lower level for weeks [22, 23]. Thus, it is possible that inhalation of CO may temporally reset an important central signal eliciting initial weight loss. However, this effect is not long lasting or is compensated for. The compensation for initial weight loss in the mice receiving 28 ppm CO is evident from the fact that these mice had significantly greater body fat compared to the HFD control group at 30 weeks which cannot be explained by differences in lean mass development, food intake or motor activity. Compensation due to alterations in central signaling or in the absorption of food in digestive track will need to be explored in future studies in which more measurements of metabolic state and hormones are made during the initial weight loss period.

## Conclusions

Our results demonstrate that CO inhalation at 200 and 28 ppm has a transient effect to prevent and reverse obesity. It is not likely that this effect is due to anorexic conditions like that seen in response to high elevation induced hypoxia as food intake was not altered by CO inhalation. While the mechanism responsible for the initial weight loss observed in the present study is not known, our results indicate that chronic inhalation of CO does not produce sustained weight loss. Other treatments to chronically increase CO levels such as CORMs appear to be more promising agents in the long term treatment of obesity.

## Abbreviations

ALDH1A1: Aldehyde dehydrogenase-1
CO: Carbon monoxide
COHb: Carboxyhemoglobin
CORMs: Carbon monoxide releasing molecules
CO-28: Carbon monoxide treatment at 28 ppm for 2 hour daily
CO-200: Carbon monoxide treatment at 200 ppm for 1 hour daily
GTT: Glucose tolerance test
HO-1: Heme oxygenase-1
IP: Intraperitoneal
NRF-1: Nuclear respiratory factor-1
PGC-1α: Peroxisomal proliferating activating receptor-γ cofactor α
ppm: Parts per million
RQ: Respiratory quotient
VCO2: Volume of carbon dioxide
VO2: Volume of oxygen
kDa: Kilodaltons.
